# Different Acupuncture Therapies for Allergic Rhinitis: Overview of Systematic Reviews and Network Meta-Analysis

**DOI:** 10.1155/2020/8363027

**Published:** 2020-04-23

**Authors:** Jinhuan Zhang, Yanying Zhang, Xingxian Huang, Kai Lan, Liyu Hu, Yirong Chen, Haibo Yu

**Affiliations:** ^1^Fourth Clinical Medical School of Guangzhou University of Chinese Medicine, Shenzhen 518033, China; ^2^Shenzhen Traditional Chinese Medicine Hospital, Shenzhen 518033, China

## Abstract

**Objective:**

To evaluate the quality of methodologies used in previous systematic reviews (SRs) and compare efficacy of different acupuncture therapies for allergic rhinitis.

**Methods:**

Seven electronic databases were searched for systematic reviews (SRs) performed on different acupuncture therapies for allergic rhinitis from inception to 15 November 2019. The AMSTAR2 instrument was employed to assess the methodological quality of included SRs. Eligible randomized controlled trials (RCTs) were selected from the included systematic reviews. We also included recent RCTs published by 15 November 2019. Cochrane risk of bias tool was utilized to determine risk of bias of the included RCTs. Pairwise meta-analyses were performed using the random-effects model. Network meta-analysis of the included RCTs was carried out using frequentist framework.

**Results:**

We identified 2 SRs with low quality and 18 SRs with very low quality, both of which contained 33 eligible RCTs (*n* = 3769). Most of these studies had unclear risk of bias. On the basis of ranking probability, NMA analysis showed that acupuncture at the sphenopalatine ganglion acupoint (OR: 1.31, 95% CI 1.07 to 1.61) had the highest probability of improving global allergic rhinitis symptoms, followed by San-Fu-Tie (OR: 1.17, 95% CI 1.08 to 1.27), manual acupuncture (OR:1.15, 95% CI 1.07 to 1.24) compared with conventional western medicine treatment. Moreover, direct comparison of the follow-up period showed that the clinical outcomes of acupuncture and related therapies at three-month (OR:1.34, 95% CI 1.17 to 1.55), six-month (OR: 1.31, 95% CI 1.10 to 1.57), and twelve-month (OR: 1.30, 95%CI 1.11 to 1.53) follow-up were better than those of traditional western medicine.

**Conclusion:**

These results indicate that for patients with allergic rhinitis who are unresponsive to conventional western medicine or cannot tolerate the side effects, acupuncture at the sphenopalatine ganglion acupoint is an effective alternative therapy. Further studies are advocated to deeply explore methodological quality of SRs by incorporating high-quality RCTs.

## 1. Introduction

Allergic rhinitis (AR) affects 10% to 20% of the of the world's population [1]. AR negatively impacts patients' social life by affecting sleep, school performance, and work productivity [2–4]. This condition also financially burdens affected individuals [5].

Acupuncture is widely used for the management of AR. The 2015 American Clinical Practice Guidelines for AR [6] and the 2018 Chinese Society of Allergy Guidelines for Diagnosis and Treatment of Allergic Rhinitis [7] recommend acupuncture for management of AR. Among the advantages cited by the American guidelines is that acupuncture offers an effective alternative therapeutic option, for the alleviation of AR symptoms, thereby improving quality of life while reducing drug prescriptions and associated side effects. However, there is limited credible evidence for its efficacy because available randomized controlled trials (RCTs) did not compare acupuncture with traditional medicinal therapy and have methodological flaws [6].

In recent years, more RCTs have compared the effectiveness of acupuncture to that of traditional medicinal therapy in allergic rhinitis. Multiple systematic reviews have documented that acupuncture therapy has higher efficacy relative to western medicine [8–10]. However, whether different acupuncture therapies are indeed superior to western medicine or even substitutes for western medicine has not been interrogated. The Chinese and American guidelines do not recommend specific types of acupuncture for AR management, and the choice of which type to prescribe is at the clinician's discretion.

Here, we conducted an overview of systematic reviews to evaluate the quality of methodology used in their preparation. We then compared the efficacy of different acupuncture therapies alone with that of western medicines through network meta-analysis. This study aimed to provide some references for clinical decision making.

## 2. Methods

### 2.1. Inclusion Criteria

SR/meta-analysis based on RCTs on the benefits of acupuncture against AR was included in the study if they met the set criteria outlined below for participants, interventions, control groups, and outcome indicators. SRs had to be in either the English or Chinese language.

#### 2.1.1. Participants

Inclusion of AR patients was not restricted by age, race, or gender. Participants were included if they had a clear AR diagnosis but were not limited to specific diagnostic criteria.

#### 2.1.2. Intervention and Comparisons

Different acupuncture protocols, including manual acupuncture, appoint catgut embedding, acupoint herb application, San-Fu-Tie, acupuncturing the sphenopalatine ganglion acupoint alone (Table 1) were included in the study. The control group was constituted as recommended by guidelines for conventional drugs including nasal corticosteroid spray, antihistamines, and immunotherapy. And it is important to note that acupoint herb application can be classified into San-Fu acupoint herb application (only applied during Sanfu period) and non-San-Fu-Tie acupoint herb application (applied without a specific time frame). In this study, acupoint herb application referred to non-San-Fu-Tie acupoint herb application.

#### 2.1.3. Outcome

To be included in an NMA, trials must include the outcome of nasal symptoms (sneezing, rhinorrhea, nasal itching, and nasal obstruction) [11], and the criteria for outcome evaluation must be clear. The outcome evaluation is also recommended for the standardization of clinical outcomes used in AR [11]. Outcomes on symptom improvements are reported on short 3-point Likert scales. On the basis of clinical observations, these short ordinal outcomes were dichotomized as “improvement” or “no improvement” in accordance with guidelines of the Cochrane handbook. In this study, the outcomes “markedly effective” and “effective” were grouped as “improvement,” and the outcome “no improvement” classified as “nonbeneficial.” Likewise, a binary assessment of global symptoms was used in the recently published systematic review of Fu et al. [10].

### 2.2. Search Strategy and Selection Criteria

**Figure 1:**
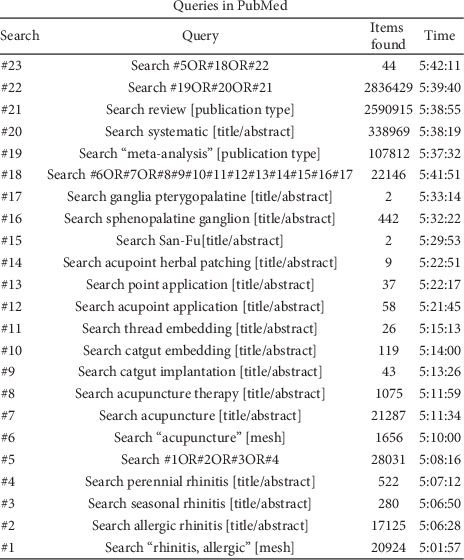
The search strategy for PubMed.

Literature analyzed in this review was searched on Cochrane library, PubMed, EMBASE, as well as on four Chinese databases (Wan Fang Digital Journals, China National Knowledge Infrastructure, Chinese Biomedical Database, and VIP Database). The searches were made for systematic reviews of acupuncture-related treatments for AR for up to November 15, 2019. A grey literature search was also done. Only publications in English or Chinese were included in this study. A typical search, as done on PubMed, is shown on Figure 1.

### 2.3. Literature Selection and Data Extraction

Two researchers (Z. J. H and H. X. X) independently screened the literature results and extracted data. Disagreements between Z. J. H and H. X. X were resolved by consulting a third researcher (Z. Y. Y). Where clarification was needed, the paper's author was contacted for more information. Literature screening involved first excluding any duplicated articles. Where articles were of high similarity, the most comprehensive one was selected. Next, the titles and abstracts were read, and any obviously irrelevant literature excluded from further analysis. Full texts of the remaining articles were then read to determine the final inclusion.

Eligible RCTs were selected from the included systematic reviews and updated RCTs that were available up to November 15, 2019. RCT screening for inclusion was done as described above, and the following information independently extracted from the RCTs by Z. J. H and H. X. X: author, year of publication, number of patients enrolled in the study, participant characteristics, duration of diseases, diagnostic criteria, duration of patient enrollment, types and uses of interventions, efficacy standard, and follow-up duration.

### 2.4. Methodological Quality and Risk of Bias Assessment

To assess the quality of methodology, we used the AMSTAR2 scale [12]. Compared with its predecessor, AMSTAR2 offered a more comprehensive assessment. AMSTAR2 takes into account 16 items that are answered “yes” and “no.” Items 2, 4, 7, 9, 11, 13, and 15 are critical fields. If the analysis reveals no item defect or only one noncritical item defect, the methodology was high quality and the SR considered accurate and comprehensive. In cases of more than one noncritical item defect, but no critical item defect, the methodology was of medium quality and the SR considered accurate. In cases of a key item defect, with or without a noncritical item defect, the methodology was considered of low quality and the SR considered not be accurate or comprehensive. In cases of more than one critical flaw, with or without noncritical item defect, the methodology was considered to be of extremely low quality and the SR considered inaccurate and incomplete [12].

The Cochrane risk of bias tool was used to estimate the risk of bias in each included RCT, which took the following into account: random sequence generation, allocation concealment, blinding of participants and personnel, blinding of outcome assessment, incomplete outcome data, selective outcome reporting, and other sources of bias. Risk of bias for each field was scored low, high, or unclear [13].

### 2.5. Data Synthesis

To execute network meta-analysis, we first performed pairwise meta-analysis, followed by network meta-analysis. Pairwise meta-analysis compared different acupuncture types relative to the control group since outcome indicators were binary. We used odd ratios (OR) and 95% confidence interval (CI) to assess the effects of different acupuncture therapies on nasal symptoms. Heterogeneity between pairwise comparisons was determined by *I*^2^ and *P* values; *I*^2^ < 25% is considered low heterogeneity, 25–50% moderate heterogeneity, and >50% high heterogeneity. *P* ≤ 0.1 was considered indicative of significant heterogeneity [14].

Random-effects network meta-analysis was done using STATA version 14.0. Network meta-analyses were executed using a network meta-analysis package [15].

The network *i* command was used to evaluate inconsistency of all comparisons.

Potential therapeutic benefits of interventions were ranked using surface under the cumulative ranking curve (SUCRA) [16].

Potential small study effects within the network were detected using comparison-adjusted funnel plots [17]. Sensitivity analysis was done by excluding studies with a sample sizes of 60 or by excluding studies with significant levels of heterogeneity.

## 3. Results

### 3.1. Literature Search Results and Study Selection

**Figure 2:**
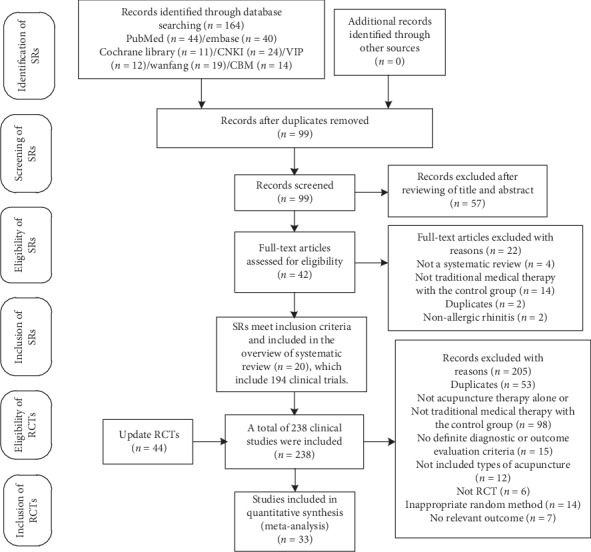
PRISMA flowchart of the study selection process.

Twenty SRs [8–10, 18–34] were selected from 99 records. The 20 SRs comprised 194 RCTs, while 44 RCTs were updated simultaneously as described for eligibility inclusion. Eventually, 33 RCTs [35–67] were included in the study. An outline of the screening procedure is shown in Figure 2.

### 3.2. Characteristics of Included RCTs

The 33 RCTs consisted of 3769 patients in studies published between 2007 and 2019. The patients included in the studies were aged between 3 and 78 years and exhibited disease course for 6 months to 36 years. The study sample size ranged from 53 to 339 patients. Treatment duration varied between 7 days and a year. Duration of follow-up ranged from 1 month to 3 years. Together, the 33 RCTs had 17 diagnostic and efficacy evaluation criteria, of which 16 were Chinese. The control groups for the 33 RCTs were all treated with western medicine, including nasal corticosteroids, antihistamines, immunotherapy alone, or their combination. Together, the 33 RCTs studied 5 types of acupuncture, including manual acupuncture (*n* = 11), acupoint herb application (*n* = 7), San-Fu-Tie (*n* = 8), acupoint catgut embedding (*n* = 5), and sphenopalatine ganglion acupoint (*n* = 2). Characteristics of the included studies are shown on Table 2.

### 3.3. Methodological Quality of Included SRs

AMSTAR 2 was used to assess the quality of methodology used in the 20 included SRs. This analysis revealed that in 3 SRs, only one critical item failed the quality test, and the quality of methodology was low. In the other SRs, more than one critical item failed the quality test and quality of methodology was very low. Among the critical items, the compliance rate on item 2 and item 4 was 10%; for item 7, it was 60%; for item 9, it was 85%; for item 11, it was 100%; for item 13, it was 95%; and for item 15, it was 70%. For the noncritical items, for items 1 and 3, it was 100%; for items 5 and 6, it was 70%; for items 10 and 16, it was 10%; for item 12, it was 95%; and for item 14, it was 80%. None of the SRs fully met the standards for item 8 (Table 3).

### 3.4. Risk of Bias among Included RCTs

**Figure 3:**
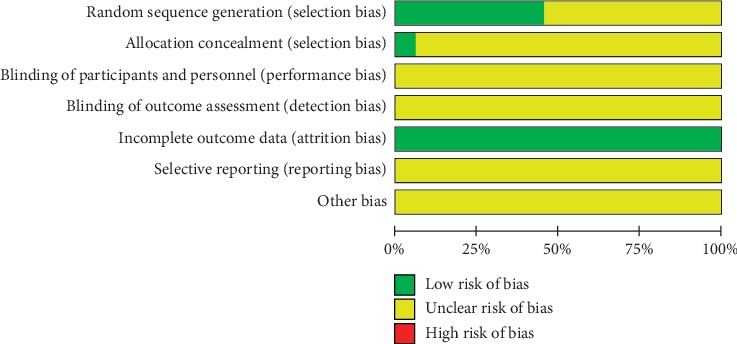
Summary risk of bias.

To minimize the risk of bias, 14 of the 33 RCTs reported using the random number table method, 1 adopted card randomization, all of which were assessed as low risk, and 15 only described randomness but did not describe the method of randomization used (these were considered unclear). In terms of allocation of concealment, only 2 trials stated opaque envelopes that were considered low risk. In terms of incomplete report outcomes, 3 trials described appropriate methods that were used in handling incomplete data and were considered low risk. The rest were not described and were judged as unclear. None of the included trials described the methods used to blind participants, implementers, and outcome evaluators, which were considered unclear risks. Because of their failure to provide protocols, the selective outcome report biases, and other biases, they were considered unclear. Details of risk bias are shown in Figure 3.

### 3.5. Results of Pairwise Meta-Analyses

We did a meta-analysis of direct comparison between five acupuncture therapies and western medicine (Table 4). Results from this analysis show that manual acupuncture, San-Fu-Tie, acupuncturing the sphenopalatine ganglion acupoint and acupoint herbal application were superior to western medicine. The acupoint catgut embedding acupuncture method did not emerge as being superior to western medicines.

### 3.6. Results of Network Meta-Analysis

#### 3.6.1. Network Plot

**Figure 4:**
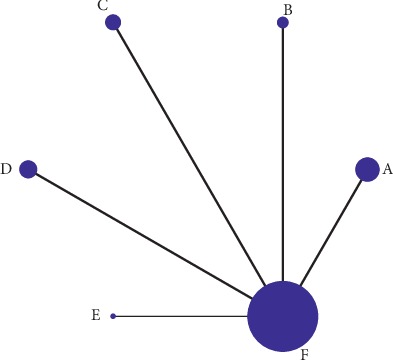
Network meta-analysis comparing the efficacy of various acupuncture methods to ameliorate AR overall symptoms relative to western medicine. Note: A, manual acupuncture; B, acupoint catgut embedding; C, acupoint herbal application; D, San-Fu-Tie; E, acupuncturing the sphenopalatine ganglion acupoint; F, western medicine.

All 33 RCTs included in our study two-arm trials. In this analysis node, size represents sample size, whereas line thickness represents the number of comparisons between two studies. This study revealed that the number of comparisons was highest between acupuncture and western medicine (*n* = 11) (Figure 4).

#### 3.6.2. Results of Comparative Effectiveness of the Six Interventions after the Treatment and Follow-Up Period

This analysis found that acupuncturing the sphenopalatine ganglion acupoint (OR: 1.31, 95% CI 1.07 to 1.61), San-Fu-Tie (OR: 1.17, 95% CI 1.08 to 1.27), and manual acupuncture (OR: 1.15, 95% CI 1.07 to 1.24) were more effective than western medicine at ameliorating the symptoms of AR (Table 5). Analysis of results from the follow-up period showed that manual acupuncture (OR: 1.63, 95% CI 1.09 to 2.44), acupoint herbal application (OR: 1.38, 95% CI 1.17 to 1.64), and acupoint catgut embedding (OR: 1.25, 95% CI 1.05 to 1.50) were more effective than western medicine at ameliorating AR symptoms. No statistically significant difference was observed between the five acupuncture treatments (Table 5).

#### 3.6.3. Results of Interventions Rank Probabilities

**Figure 5:**
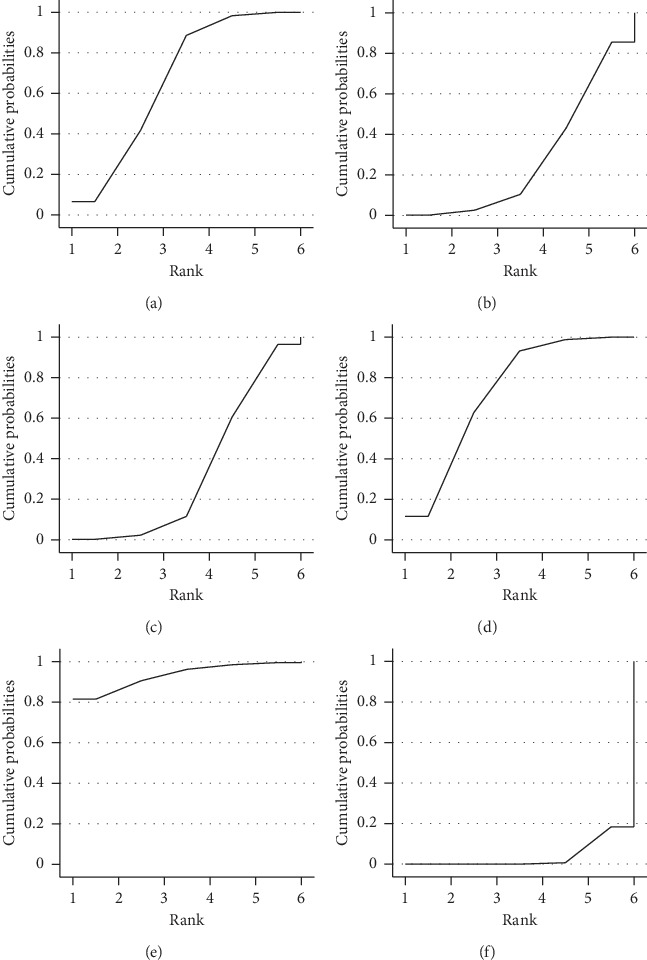
SUCRA for improving overall symptoms of AR. Note: A, manual acupuncture; B, acupoint catgut embedding; C, acupoint herbal application; D, San-Fu-Tie; E, acupuncturing the sphenopalatine ganglion acupoint; F, western medicine.

The cumulative area of SUCRA represents the probability of each intervention ameliorating the symptoms of AR (Figure 5). This analysis revealed that acupuncturing at the sphenopalatine ganglion acupoint (92.4%) exhibits the highest probability of improving AR symptoms, followed by San-Fu-Tie (82.8%), acupuncture (62.2%), acupoint herb application (34.4%), acupoint catgut embedding (24.8%), and western medicine (3.3%).

#### 3.6.4. Sensitivity Analysis and Small Sample Effect

**Figure 6:**
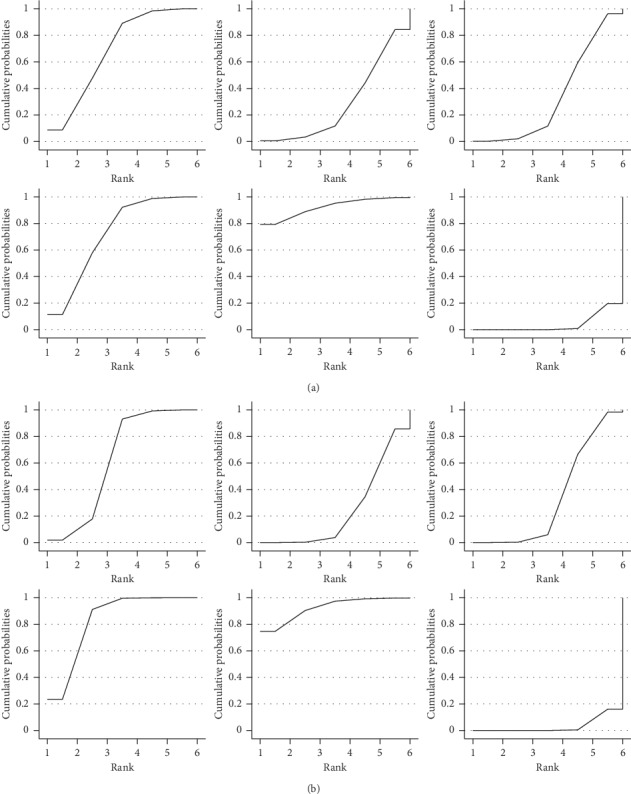
(a) Excluding 2 studies whose sample size was smaller than 60; (b) excluding study that exhibited a significant level of heterogeneity. Sensitivity analysis: SUCRA for ameliorating overall symptoms of AR. Note: A, manual acupuncture; B, acupoint catgut embedding; C, acupoint herbal application; D, San-Fu-Tie; E, acupuncturing the sphenopalatine ganglion acupoint; F, western medicine.

We performed sensitivity analysis in two ways, one by excluding 2 studies [47, 60] whose sample size was smaller than 60. Results of this analysis indicated that exclusion of the 2 studies did not alter the ranking established earlier (Figure 6(a)); SUCRA—E (92.3%) > D (72.1%) > A (68.8%) > C (33.9%) > B (28.8%) > F (4.1%). The other sensitivity test was done by excluding study [64] that exhibited a significant level of heterogeneity, which too, did not alter the ranking (Figure 6(b)); SUCRA—E (92.6%) > D (72.4%) > A (68.4%) > C (33.8%) > B (28.7%) > F (4.0%).

**Figure 7:**
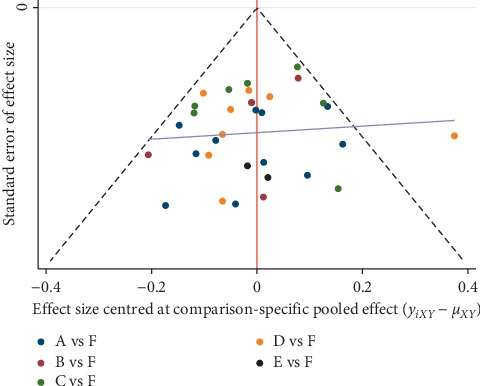
Comparison-adjusted funnel plots for improving overall symptoms of AR. Note: A manual acupuncture; B acupoint catgut embedding; C acupoint herbal application; D San-Fu-Tie; E acupuncturing the sphenopalatine ganglion acupoint; F western medicine.

**Figure 8:**
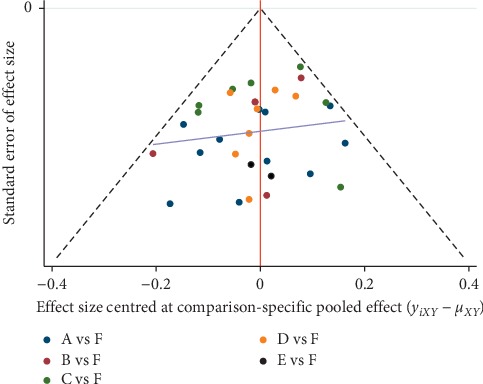
Sensitivity analysis: comparison-adjusted funnel plots for improving overall symptoms of AR. Note: A manual acupuncture; B acupoint catgut embedding; C acupoint herbal application; D San-Fu-Tie; E acupuncturing the sphenopalatine ganglion acupoint; F western medicine.

An adjusted funnel plot analysis revealed a symmetrical funnel plot and no obvious small sample effects (Figure 7). However, the distribution scattered one point outside of the funnel plot. The point scattered out of the funnel plot represented a heterogeneous study [64]. Exclusion of this study resulted in scattering of all the points inside the funnel plot (Figure 8).

### 3.7. Follow-Up Efficiency

Analysis of the follow-up data revealed that 7 trials (20.6%) reported effective rates. Of these, 1 trial reported effective rates after 1 month of follow-up, 4 reported effective rates after 3 months of follow-up, 3 reported effective rates after 6 months of follow-up, and 3 trials reported effective rates after 12 months of follow-up. With the exception of the 1-month follow-up, the follow-up rates for 3, 6, and 12 months indicated that acupuncture therapy was superior to western medicine group (Table 6).

### 3.8. Adverse Effects and Loss to Follow-Up

#### 3.8.1. Adverse Events

Four (4) trials, 1 on manual acupuncture, 1 on San-Fu-Tie, and 2 on acupoint catgut embedding, reported adverse outcome. The 4 reported symptoms of the adverse reactions alleviated after discontinuation of the treatment or after a few days' rest. However, none reported serious adverse events occurred. Specifically, Huang[39] reported that 8 cases receiving western medicine complained of drowsiness and fatigue, 1 case complained of joint pain and fatigue, and 1 case complained of slight rash. Symptoms stopped on discontinuation of oral loratadine treatment. In the acupuncture group, 6 case patients complained of improper pressure during needle extraction, resulting in slight subcutaneous hematoma that cleared naturally within days.

Liu [46] reported 2 cases in the acupoint embedding treatment group who had mild adverse reactions, and one who had bruises after acupoint treatment. These effects were considered to have been caused by damaged subcutaneous capillaries, and the symptoms resolved after hot compress. On the day of treatment, a patient complained that their left arm was not flexible, and this was considered to have been caused by a strong sense in hegu acupoint. The symptoms resolved within 3 days. One patient treated with western medicine presented with a nasal cavity and a small amount of blood in the nose after one month of treatment, but during follow-up, it was self-reported that the symptoms had resolved.

Feng [47] reported 2 cases of adverse reactions in both acupoint embedding and western medicine groups. One case treated with acupoint embedding exhibited induration at the embedding acupoint while another patient reported fainting during the acupuncture procedure. In the western medicine group, one patient was allergic to medicine and another complained of drowsiness. This paper did not provide treatment or prognosis details.

Wang [54] reported one case that suffered skin irritation after San-Fu-Tie treatment and 11 cases of adverse reactions in the western medicine treated group, including 3 cases of mild nosebleeds, 2 cases of drug dependence, 4 cases of dry nasal cavity, and 2 cases of perforated nasal septum. The difference between the two groups was statistically significant (*χ*2 = 9.470, *P*=0.004).

#### 3.8.2. Dropout Patients

Three trials reported patient dropout. Liang et al. [50] reported that 15 patients receiving acupuncture dropped out of the study. Of these, 1 dropped out due to dizziness during treatment, 4 dropped out for personal reasons, and 10 had not received treatment as prescribed. In the western medicine-treated group, 15 cases dropped from the study. Among them, 6 withdrew from the study by themselves, and 9 cases were not treated as prescribed.

Liu [46] reported that 2 patients receiving acupoint embedding acupuncture dropped out of the study, 1 for work-related reasons, and the other because of protocol violation. Among the western medicine-treated group, 2 cases dropped out (one because of protocol violation and the other one elected to stop treatment).

Wang et al. [62] reported that 1 case in the acupuncture-treated group and 2 cases in the western medicine-treated dropped out of the study due to incomplete compliance with the test requirements.

Overall, the dropout rate between the two groups is balanced and mainly caused by violation of the treatment plan or moving, rather than poor efficacy.

## 4. Discussion

In this study, we used AMSTAR2 to evaluate the quality of methodology used in 20 SRs. Our analysis shows that 85% of the SRs had very low quality of methodology, which lowers credibility of the conclusions by drawn in the systematic reviews that acupuncture is superior to western medicine. In terms of ameliorating the symptoms of AR, the 33 RCTs that we included in our direct comparison analysis indicated that the 4 types of acupuncture therapy have a superior performance relative to western medicine. However, in the direct comparison of San-Fu-Tie and compared western medicine, a heterogeneity of 60.9% was observed. After a sensitivity analysis, 1 study [64] was withdrawn, and the heterogeneity fell to 0. This manipulation did not change the outcome of the earlier analysis, indicating that the results were robust. Among the 8 trials in San-Fu-Tie was compared with western medicine, only 1 study [64] indicated a relatively good efficacy, although this was not statistically significant. San-Fu-Tie can therefore still be regarded as superior to western medicine. In NMA, ranking on the basis of improved AR symptoms identified acupuncturing the sphenopalatine ganglion acupoint as the best treatment and western medicine as the worst. Subsequent sensitivity analysis did not significantly change the ranking results.

This study assessed the comparison between the efficacy of the 6 interventions at improving AR during the follow-up period and found acupuncture to still be better than western medicine after 12 months of follow-up. In terms of adverse events, acupunctural therapies do not show overt adverse reactions. Therefore, patients who are intolerant to western medicine or interested in alternative therapy can consider acupunctural procedures.

Our results are partly consistent with those from previous studies. Fu [10] showed that acupuncture of the sphenopalatine ganglion points is better than other approached, including sham needles, western medicine, and conventional acupuncture points. However, there was no significant difference and obvious heterogeneity (RR = 1.18, 95% CI [0.88, 1.57], I2 = 93%) between acupuncturing the sphenopalatine ganglion acupoint and western medicine alone. The reason is that 2 studies evaluated by the authors did not report clear diagnostic criteria or their efficacy criteria and were therefore excluded from our study. A systematic review by Zhang et al. [9] showed that acupuncture alone (OR = 2.42, 95% CI 1.15 to 5.09) *I*^2^ = 2%) was better than western medicine therapy. A study by Zhou et al. [34] showed that there was no statistical difference between acupuncture alone and western medicine within less than 3 months (OR = 1.04, 95% CI 0.92 to 1.17), but acupoint application plus western medicine was better than western medicine alone (OR 1.22, 95% CI 1.12 to1.33). Interestingly, after treatment for beyond 3 months, acupoint application alone appears better than western medicine, and this might be related to the better prognosis of acupoint application. To the best of our knowledge, no systematic review has analyzed San-Fu-Tie and western medicine alone. A review by Xie et al. [8] shows that acupoint catgut embedding alone is better than western medicine. However, our review of this report revealed studies with ambiguous diagnosis, and incorrect random methods were included, all of which were excluded in our study.

Acupuncture is traditional medicine method that can balance Yin and Yang and improve physique at a high efficiency. The mechanisms of its effects are progressively becoming clearer. Research shows that acupuncture can reduce immunoglobulin [68], regulate Th1/Th2 levels [69], inhibit the release of inflammatory mediators [70], and reduce inflammatory neuropeptides [71].

Acupuncturing the sphenopalatine ganglion acupoint, combined with the theory of traditional Chinese and western medicine, was first reported in 1990 [72]. In this approach, acupuncture is used to stimulate the sphenopalatine ganglion with the aim of adjusting the balance between sympathetic and parasympathetic functions. The mechanism of acupuncture action the sphenopalatine ganglion in the treatment of AR includes 1) the stimulation of the sympathetic nerve fibers in the distribution area of this ganglion, which causes blood vessel constriction, reduced blood flow to the nasal mucosa and cavernous body, reduced glandular secretion, widening of the nasal passage, and reduced the turbinate, thereby improving ventilation and ameliorating associated symptoms, and (2) through stimulation of central nervous autonomic control. It is hypothesized that after the nerve ganglion is stimulated, the stimulation is reflected on the central system, which in turn stimulates bilateral nerves and therefore simultaneously regulates bilateral nasal mucosa [73–74].

One limitation of our study is that it did not include indirect comparisons and placebos. These were excluded because our aim was to establish which acupunctural procedures are better alternatives to western medicine. In addition, the types of western medicine are not studied separately. This is mainly because there are many kinds of such treatments with various applications. These will be the focus of future studies.

Second, only two of the included SRs were rated of low quality of methodology while the methodologies of remainder were considered to be of extremely low quality. This is attributable to the fact that in the literature search of key items, most of the studies were not registered in advance, and grey literature was ignored in literature searches. Future SRs reporting standards should adhere to the requirements of PRISMA and make improvements to draw more rigorous conclusions [75].

Third, the RCT methodology of included studies was generally poor since most of the risk items are unclear, particularly allocation concealment, blindness, and selective outcome reporting. This limitation might lower credibility of our conclusions. We recommend that in future, researchers follow CONSORT reporting norms [76]. With regard to implementation of blind method, because the reported outcomes in this study are subjective symptom scores, third-party evaluators may implement blind methods so as to reduce bias and enhance reliability of the results.

Fourth, the included RCTs are from China, and there may be differences in diagnostic criteria and outcome evaluation criteria between studies. Because the report of acupuncture use is not sufficiently detailed, it may cause limitations in the recommendation of conclusions. In this study, we have attempted to describe acupoints and usage methods in as much detail as possible in the feature table. We recommend that future acupuncture clinical trials follow the STRICTA standard [77] and be done in as many countries and regions as feasible.

## 5. Conclusion

Our findings indicate that acupuncturing the sphenopalatine ganglion acupoint is a viable alternative for patients with allergic rhinitis and are unresponsive to conventional western medicine or cannot tolerate its side effects. We recommend that future acupuncture systematic reviews put more emphasis on the quality of methodologies used in the underlying studies and that high-quality RCTs be undertaken to enhance the reliability of the conclusions drawn.

## Figures and Tables

**Table 1:** Definitions of different acupuncture therapies.

Type of acupuncture	Definitions
Manual acupuncture	A needle is inserted into a specific acupoint at a certain angle according to the theory of traditional Chinese medicine. Acupuncture techniques such as twisting and lifting are used to stimulate specific parts of the body to treat diseases
Appoint catgut embedding	This approach involves implantation of a sheep gut or other absorbable thread to acupuncture points in line with the theory of acupuncture and meridian. The medical sheep gut is buried into the corresponding acupoint area to treat diseases
Acupoint herb application	In this type of acupuncture, a special Chinese medicine is applied to specific acupoints on the human body. This method produces both acupoint stimulation and pharmacological effects.
San-Fu-Tie	San-Fu-Tie is a plaster that is applied on the dog days. “San-Fu” is a collective term for first, second, and third dog day, and it is the hottest season of the year
Acupuncturing at sphenopalatine ganglion acupoint	Acupuncture at the sphenopalatine ganglion acupoint, combined with the theory of traditional Chinese and western medicine. This acupuncture is used to stimulate the sphenopalatine ganglion, to balance sympathetic and parasympathetic functions

**Table 2:** Characteristics of included studies.

First author, year of publication	Patient admission time	AR diagnostic and outcome evaluative criteria	Number T/C (M/F)	Age (range/mean± SD) (T/C)	Course of the disease (range/mean± SD) (T/C)	Types of intervention	Acupoint selection of intervention	Intervention (frequency and treatment duration)	Control (dosage on prescription and treatment duration)	Outcome observation time	Outcome measure
Chen et al., 2015 [35]	From May 2013 to December 2013	A1/A7	(17/17)/(14/18)	(40 ± 9) y/(40 ± 1) y	(6.8 ± 4.4) y/(7.1 ± 4.3) y	MA	GV20, EX-HN3, LI20, LI4, LR3, BL18, BL20, BL13, BL23, DU14	20 min, 3 times per week for 8 weeks	Cetirizine, 10 mg, qd, 8 w	After treatment finished	Total effective rates

Fang and Shi, 2015 [36]	NR	A1/A6	(16/15)/(15/16)	(39 ± 16) y/(42 ± 14) y	(5.12 ± 3.50) y/(6.80 ± 4.15) y	MA	EX-HN3, LI20, GB20, DU14, DU13, DU12, LI4, ST36	30 min, 5 times per week for 4 weeks	Loratadine, 10 mg, qd, 4 w	After treatment finished	Total effective rates

Ou et al., 2014 [37]	From July 2010 to January 2013	A4/A4	(12/21)/(10/23)	(29 ± 3) y/(28 ± 4) y	(6.6 ± 3.1) y/(5.80 ± 4.1) y	MA	EX-HN8, EX-HN3, LI20 plus point selection treatment based on syndrome differentiation	30 min, once a day, 0 times a course, two courses, 3–5 days' rest between the two courses	Desloratadine dry suspension, 5 mg, qd, 20 d	After treatment finished	Total effective rates

Xia et al., 2012 [38]	From October 2008 to March 2012	A1/A5	(20/37)/(28/27)	35.2 y/36.4 y	2.02 y/1.99 y	MA	LI20, EX-HN8, EX-HN3, LI4 plus point selection treatment based on syndrome differentiation	20 min, 3 times per week for 4 weeks	Cetirizine, 10 mg, qd, 4 w	After treatment finished	Total effective rates

Huang, 2012[39]	NR	A5/A5	(13/21)/(15/21)	(36.54 ± 16.2) y/(37.87 ± 15.68) y	(10.79 ± 5.58) y/(9.87 ± 6.35) y	MA	LI20, LI4, ST36	30 min, 20 days	Cetirizine, 10 mg, qd, 4 w	After treatment finished	Total effective rates, adverse effects

Zhang, 2012 [40]	NR	A1/A6	(13/17)/(15/15)	(10.25 ± 2.56) y/(11.12 ± 2.12) y	NR	San-Fu-Tie	DU14, bilateral (BL13, BL20, LI4)	In the annual “Sanfu” and “Sanjiu” apply, a year 6 times	Dermatophagoides farinae drops, sublingual, use 1–4 drops successively, 1 year	After treatment finished	Total effective rates

Shi, 2011 [41]	NR	A6/A6	(15/15)/(18/12)	(20–56) y/(19–58) y	2y-6 y	AHP	DU14, bilateral (BL13, BL17, ST36)	Keep patching 3–6 h, 15 days	Cetirizine, 10 mg, qd, 15 days	After treatment finished	Total effective rates

Mi and Yu, 2011 [42]	NR	A6/A6	49/44	(37.14 ± 13.63) y	(4.41 ± 2.30) y	AHP	BL13, BL21, BL20, BL12, BL43, BL23, EX-B1, BL15	Once every 10 days, keep patching 1-2 h, 9 times	Budesonide nasal spray (rhinocort), each nostril 1 spray, bid, 60 days	After treatment finished and 3 months after the end	Total effective rates

Wu et al., 2005 [43]	NR	A4/A4	179/160	(3–71) y	(1–35) y	AHP	DU14, BL12	Once every 10 days, keep patching 10 h, 4 times	Desensitization treatment	After treatment finished and relapse rate after 2 months of treatment	Total effective rates

Zhuang and Wu, 1997 [44]	NR	A4/A6	106/94	(3–71) y	(1–35) y	AHP	DU14, BL12	Once every 10 days, keep patching 10 h, 4 times	Desensitization treatment	After treatment finished	Total effective rates

Gu et al., 2012 [45]	NR	A6/A6	34/46	(10–72) y	1 y–32 y	ACE	EX-HN3, LI20, EX-HN8, DU14, BL13, BL23, LI11, ST36	Once every 15 days, 4 times	Cetirizine, 10 mg, qd, 28 d	After treatment finished	Total effective rates

Liu, 2012 [46]	From October 2011 to January 2012	A5/A5	(10/2)/(12/20)	NR	(5.72 ± 4.62) y/(5.25 ± 3.40) y	ACE	(First, LI20, EX-HN3, LI4, Second, LI11, ST36)	Once every 2 weeks for 4 weeks	Triamcinolone acetonide nasal spray, each nostril 2 spray, bid, 4 w	After treatment finished	Total effective rates, adverse effects

Feng, 2008 [47]	NR	A5/A5	(10/18)/(9/16)	(18–56) y/(19–56) y	(1–10) y	ACE	SI19, BL13, ST36	Once every ten days, 6 times	Loratadine, 10 mg, qd, 42 days	After treatment finished and 3 months after treatment finished	Total effective rates, adverse effects

Huang, 2012 [48]	From December 2008 to October 2010	A9/A2	100/100	NR	NR	San-Fu-Tie	BL13, BL20, ST36	Sanfu, first day of each fu/Keep patching 3–4 h, 3 fus	Beclomethasone dipropionate aqueous nasal spray, each nostril 2 spray, bid-tid, ten weeks	After treatment finished	Total effective rates

Kong, 2010 [49]	From May 2006 to October 2009	A12/A2	(32/18)/(30/20)	35.3/36	6.5 y/6.8 y	San-Fu-Tie	BL11, BL12, BL20, BL43, BL13, DU9	Sanfu, first day of each fu/keep patching 4–6 h, 3 fus	Cetirizine, 10 mg, qd, 15 days	After treatment finished	Total effective rates

Liang et al., 2019 [50]	From June 2016 to June 2018	A1/A5	(29/31)/(28/32)	(38.92 ± 12.86)y/(42.77 ± 11.13)y	(4.88 ± 6.61) y/(4.58 ± 5.83) y	MA	EX-HN8, EX-HN3, LI20	40 minutes , once every other day, 10 times	Loratadine, 10 mg, qd, 20 days	After treatment finished	Total effective rates, adverse effects

Li et al., 2019 [51]	From March 2017 to May 2018	A10/A11	(20/17)/(19/19)	(36.65 ± 2.19) y/(38.35 ± 2.31) y	(10.43 ± 1.08) m/(10.84 ± 1.05) m	MA	EX-HN9, Bi qiu	Once a day for 20 min each time for 14 days	Loratadine, 10 mg, qd, 14 days	After treatment finished	Total effective rates

Feng et al., 2018 [52]	From March 2015 to October 2017	A1/A5	(16/20)/(15/20)	30.3 y/30 y	5.4 y/5.3 y	SG	Sphenopalatine ganglion	Once a week for 4 weeks	Cetirizine, 10 mg, qd, 4 weeks	After treatment finished	Total effective rates

Wang and Leng, 2017 [53]	From December 2014 to December 2015	A13/A5	(29/21)/(15/21)	(37.11 ± 8.34) y/(36.54 ± 7.42) y	(3.76 ± 1.04) y/(4.02 ± 1.13) y	MA	EX-HN8, LI4, LI20, DU23, ST36	30 minutes	Loratadine, 10 mg, qd, 4 weeks	After treatmentfinished	Total effective rates

Wang, 2016 [54]	From 2012 to 2013	A14/A8	(26/24)/(27/23)	(42.3 ± 5.5) y/(41.4 ± 5.6) y	(5.3 ± 1.5) y/(5.4 ± 1.2) y	San-Fu-Tie	DU14, BL13, BL43, BL23, RN17	Sanfu, first day of each fu/keep patching 4–6 h, 3 fus	Beclometasone dipropionate aqueous nasal spray, each nostril 1 spray, bid,	After treatment finished	Total effective rates, adverse effects

Liu, 2014 [55]	NR	A1/A2	(29/21)/(27/23)	(6.2 ± 1.3) y/(6.8 ± 1.8) y	(11.0 ± 3.0) m/(12.0 ± 3.1) m	AHP	BL13, BL17, RN17	Keep patching 12 hours and 3 days, once every 2 weeks, 3 times	Cetirizine syrup, qd, 6 weeks	After treatment finished	Total effective rates

Wei, 2019 [56]	From June 2016 to October 2017	A1/A1	(16/14)/(18/12)	(42 ± 8.31)y/(40 ± 10.82)y	(14.28 ± 31.46)m/(13.57 ± 29.45)m	ACE	Sphenopalatine ganglion and superior cervical ganglion, EX-HN8, BL13, LI11, ST36	Once every 2 weeks, 2 times	Loratadine, 10 mg, qd, 4 weeks	After treatment finished	Total effective rates

Xie, 2018 [57]	From January 2017 to December 2017	A15/A5	(26/14)/(22/18)	NR	NR	ACE	BL13, BL20, BL23, BL17, ST36, SP10	Once every 3 weeks, 4 times	Cetirizine, 10 mg, qd, 12 weeks	After treatment finished	Total effective rates

Wen, 2013 [58]	NR	A5/A5	(26/14)/(22/19)	(25.3 ± 0.32) y/(27.2 ± 0.29) y	(8.5 ± 0.21) y/(7.3 ± 0.24) y	ACE	LI20, CV12, ST25, ST36 plus point selection treatment based on syndrome differentiation	Once every 15 days, 4 times	Loratadine, 10 mg, qd, 2 months	After treatment finished and 1 month and 3 months after treatment finished	Total effective rates

Cao et al., 2011 [59]	NR	A4/A6	80/80	(4–73) y	1 y–36 y	San-Fu-Tie	EX-B1, BL13, BL43, CV22, CV17	Sanfu, first day of each fu/once per fu, once every 7 days, keep patching 0.5–4 h, 3 fus	Ebastine tablets, 10 mg, qd and beclometasone dipropionate aqueous nasal spray, each nostril 2 spray, tid, 10 days	After treatment finished and 3 years after treatment finished	Total effective rates

Miao, 2015 [60]	From January 2006 to February 2013	A16/A16	(12/16)/(9/17)	(8–51) y	NR	MA	LI20 (left), EX-HN8 (right), GB20 (left), LI4 (right), BL23 (left), BL13 (right), ST36 (left), (on both sides of alternating)	Once a day, 6 times a week for 4 weeks	Loratadine, 10 mg, qd plus azolastine nasal spray, two to three drops, four to five times a day, 4 weeks	After treatment finished and relapse rate after 1 year of treatment finished	Total effective rates

Wang et al., 2014 [61]	From July 2011 to September 2013	A2/A2	(62/70)/(56/60)	43.7 y/45.1 y	(0.5–31) y	AHP	BL13, BL12, BL20, DU14	Sanfu, first day of each fu/Once per fu, once every 10 days, middle fu, end fu of twice, keep patching 4–6 h, 3 fus	Loratadine, 10 mg, qd, 6 days,	After treatment finished	Total effective rates

Wang et al., 2013 [62]	From March 2011 to December 2012	A6/A6	(19/22)/(17/23)	(44 ± 10) y/(40 ± 10) y	(7.9 ± 5.3) y/(6.6 ± 5.0) y	MA	DU20passDU21, DU23passDU24, EX-HN3 pass Shangen, LI20 pass Shangen	30 minutes, 3 times a week for 4 weeks	Loratadine, 10 mg, qd and sodium cromoglycate eye drops, each nostril 2 spray, bid, 12 days	After treatment finished and 3 months after treatment finished	Total effective rates, adverse effects

Xu, 2015 [63]	NR	A12/A2	(14/16)/(17/13)	43 y/43y	12.5 y/16.5 y	MA	LI20, EX-HN8, BL7, EX-HN3, LU7, LI4, plus point selection treatment based on syndrome differentiation	20 minutes, once a day for 1 month.	Loratadine, 10 mg, qd, 1 months	After treatment finished	Total effective rates

Xu 2012 [64]	From July 2008 to September 2010	A5/A3	(20/25)/(22/23)	30.6 y/32.7 y	4.2 y/4.5 y	San-Fu-Tie	DU14, BL43, BL13, BL20, BL23	Sanfu, first day of each fu/once per fu, once every 10 days, keep patching 8 h, 3 fus	Clarityne10 mg, qd, and budesonide nasal spray (rhinocort), each nostril 1 spray, bid, 7 days	1 month and 1 year after treatment finished	Total effective rates

Wen et al. 2007 [65]	From June 2003 to May 2005	A6/A6	(62/56)/(60/58)	35.6 y/34.3 y	3.8 y/3.6 y	AHP	DU14, BL13, BL20, BL23	Once per 5–7 days, keep patching 4–6 h, 6 times	BCG polysaccharide nucleic acid (BCG-PSN), 1 mL, qd, im, 10 days	1 month, 6 month, 12 month after treatment finished	Total effective rates

Liu, 2011 [66]	From July 2006 to August 2010	A5/A5	(78/74)/(77/73)	(8.25 ± 4.3) y/(8.16 ± 4.43) y	(0.5–14) y	San-Fu-Tie	BL13, LU7, DU14, BL10, BL20	Sanfu, first day of each fu/keep patching 3-4 h, 3 fus	Clarityne, 10 mg, qd, 2–4 weeks	3 months (only for recurrence rate) after treatment finished	Total effective rates

Hu and Sun 2010 [67]	From July 2006 to September 2008	A5/A5	(37/23)/(31/29)	(8–69) y	(0.5–9) y	San-Fu-Tie	BL43, DU14, BL12, BL13, EXHN15, BL20, BL17, BL23	Sanfu, first day of each fu/once per 10 days, keep patching 2–6 h, 3 times	Cetirizine AQ(Zyrtec), 10 mg, qd. Beclomethasone dipropionate aqueous nasal spray(Beconase), 2 sprays daily, tid, 10 days	After treatment finished	Total effective rates

A1, Guidelines for the diagnosis and treatment of allergic rhinitis (2009, Wuyi mountain); A2, the State Administration of Traditional Chinese Medicine (1994); A3, otolaryngology head and neck surgery (2006); A4, diagnostic criteria and efficacy evaluation criteria for allergic rhinitis, Otolaryngology Branch of Chinese Medical Association (1998); A5, Principles and Recommendations for the Diagnosis and Treatment of Allergic Rhinitis (Lanzhou, 2004); A6, Diagnostic Criteria for Diagnosis and Therapeutic Evaluation of Allergic Rhinitis (revised in 1997, Haikou); A7, total nasal symptom score and nimodipine method; A8, the State Administration of Traditional Chinese Medicine (1997); A9, Allergic Rhinitis and Its Impact on Asthma (ARIA, 2008, WHO); A10, Guidelines for the diagnosis and treatment of allergic rhinitis (2015, Tianjin); A11, The state administration of traditional Chinese medicine (2010); A12, Practical science of otolaryngology (1998); A13, Otolaryngology in traditional Chinese medicine (2007); A14, Guidelines for the diagnosis and treatment of allergic diseases (2013); A15, Guidelines for the diagnosis and treatment of allergic rhinitis, Otolaryngology Branch of Chinese Medical Association (2010); A16, the State Administration of Traditional Chinese Medicine (1995). MA: manual acupuncture; SG: sphenopalatine ganglion; ACE: acupoint catgut embedding; AHP: acupoint herbal patching; y: year, m: month; w: week; d: day; h: hour. NR: not reported, M: man, F: female. BCG, Bacillus Calmette-Guerin; C = control, I = intervention; qd: quaque die; bid: bis in die; tid: ter in die.

**Table 3:** Methodological quality of included systematic reviews.

First author and year	AMSTAR2 item															
	1	2^*∗*^	3	4^*∗*^	5	6	7^*∗*^	8	9^*∗*^	10	11^*∗*^	12	13^*∗*^	14	15^*∗*^	16
Li et al., 2018 [18]	Y	N	Y	PY	Y	Y	N	PY	Y	N	Y	Y	Y	N	Y	N
Liu et al, 2017 [19]	Y	N	Y	PY	Y	Y	N	PY	Y	N	Y	Y	Y	N	Y	N
Zhang et al., 2017 [9]	Y	N	Y	PY	Y	Y	N	PY	Y	Y	Y	Y	Y	Y	N	N
Qu and Liu, 2016 [20]	Y	N	Y	Y	N	N	N	PY	Y	N	Y	Y	Y	Y	Y	N
Lin and Lim, 2017 [21]	Y	N	Y	PY	Y	Y	Y	PY	Y	N	Y	N	N	Y	Y	N
Zhao et al., 2009 [22]	Y	N	Y	PY	N	N	N	PY	N	N	Y	Y	Y	N	Y	N
Chen, 2015 [23]	Y	N	Y	PY	Y	Y	Y	PY	Y	N	Y	Y	Y	Y	Y	N
Huang, 2013 [24]	Y	N	Y	PY	Y	Y	Y	PY	Y	N	Y	Y	Y	Y	Y	N
Li et al., 2013 [25]	Y	N	Y	Y	Y	Y	Y	PY	Y	Y	Y	Y	Y	Y	Y	N
Wang, 2016 [26]	Y	N	Y	PY	Y	Y	Y	PY	Y	N	Y	Y	Y	N	N	N
Xiao et al., 2009 [27]	Y	N	Y	PY	Y	Y	Y	PY	Y	N	Y	Y	Y	Y	N	N
Xie et al., 2018 [8]	Y	N	Y	PY	N	N	N	PY	Y	N	Y	Y	Y	Y	N	N
Liang, 2015 [28]	Y	N	Y	PY	N	N	Y	PY	N	N	Y	Y	Y	Y	Y	N
Liang et al., 2015 [29]	Y	N	Y	PY	N	N	Y	PY	N	N	Y	Y	Y	Y	Y	N
Chen et al., 2016 [30]	Y	N	Y	PY	N	N	Y	PY	Y	N	Y	Y	Y	Y	Y	N
Zeng, 2017 [31]	Y	N	Y	PY	Y	Y	Y	PY	Y	N	Y	Y	Y	Y	N	N
Liu et al., 2016 [32]	Y	N	Y	PY	Y	Y	N	PY	Y	N	Y	Y	Y	Y	Y	N
Zhang, 2012 [33]	Y	N	Y	PY	Y	Y	N	PY	Y	N	Y	Y	Y	Y	N	N
Fu et al., 2019 [10]	Y	Y	Y	PY	Y	Y	Y	PY	Y	N	Y	Y	Y	Y	Y	Y
Zhou et al., 2016 [34]	Y	Y	Y	PY	Y	Y	Y	PY	Y	N	Y	Y	Y	Y	Y	Y
Percentage of Y	100	10	100	10	70	70	60	0	85	10	100	95	95	80	70	10

^*∗*^Critical items; Y: yes; N: no. PY: partial yes; Item 1: Did the research question and inclusion criteria include PICO? Item 2: Was there a prepublished plan? Were there significant biases in research and protocols? Item 3: Did the author explain the type of study design included? Item 4: Was a comprehensive literature search strategy used?. Item 5: Were repeated research screenings performed? Item 6: Was there duplicate data extraction? Item 7: Was there a list of excluded studies with reasons for exclusion given? Item 8: Was there a list of included studies with reasons for inclusion given? Item 9: Were appropriate tools used to assess the risk of bias for each included study? Item 10: Were the sources of funding disclosed in the study report? Item 11: If a meta-analysis was performed, were the results statistically combined using appropriate methods? Item 12: If a meta-analysis was performed, was the effect of the risk of bias explained in the results? Item 13: If a meta-analysis was performed, was the effect of the risk of bias explained in the discussion? Item 14: Was heterogeneity properly explained in the discussion? Item 15: If quantitative analysis was performed, were publication biases adequately investigated and their possible impacts discussed? Item 16: Were any potential sources of conflicts of interest disclosed?

**Table 4:** Pairwise meta-analysis of effectiveness for improving overall symptoms of allergic rhinitis.

Comparison	Study number	OR (95% CI)	*P*	*I* ^2^ (%)	*z*
A VS F	11	1.16 (1.09, 1.24)	0.000	11.6	4.54
B VS F	5	1.08 (1.00, 1.17)	0.066	36.5	2.04
C VS F	7	1.08 (1.02, 1.13)	0.006	49.80	2.74
D VS F	8	1.20 (1.12, 1.27)	0.000	60.9	2.54

Note: A, manual acupuncture; B, acupoint catgut embedding; C, acupoint herbal application; D, San-Fu-Tie; F, western medicine.

**Table 5:** Network meta-analysis comparisons of effectiveness for improving overall symptoms of allergic rhinitis.

E	1.20 (0.81, 1.78)	0.79 (0.49, 1.28)	0.93 (0.68, 1.27)	1.03 (0.75, 1.41)	1.29 (0.99, 1.67)
1.12 (0.90, 1.39)	D	0.66 (0.40, 1.08)	0.77 (0.55, 1.08)	0.85 (0.61, 1.20)	1.07 (0.80, 1.43)
1.14 (0.92, 1.41)	1.02 (0.91, 1.13)	A	1.18 (0.76, 1.83)	1.30 (0.84, 2.02)	**1.63 (1.09, 2.44)** ^*∗*^
1.23 (0.99, 1.52)	1.10 (0.98, 1.22)	1.08 (0.97, 1.20)	C	1.10 (0.86, 1.41)	**1.38 (1.17, 1.64)** ^*∗*^
1.25 (0.99, 1.56)	1.11 (0.98, 1.26)	1.09 (0.97, 1.24)	1.01 (0.90, 1.15)	B	**1.25 (1.05, 1.50)** ^*∗*^
**1.31 (1.07, 1.61)** ^*∗*^	**1.17 (1.08, 1.27)** ^*∗*^	**1.15 (1.07, 1.24)** ^*∗*^	1.07 (0.99, 1.15)	1.05 (0.96, 1.16)	F

Note: A, manual acupuncture; B, acupoint catgut embedding; C, acupoint herbal application; D, San-Fu-Tie; E, acupuncturing the sphenopalatine ganglion acupoint; F, western medicine. The values in the lower triangle and upper triangle of the table suggest the OR of the column index compared with that of the row index. The lower left is the effective rate at the end of treatment, and the upper right is the effective rate at follow-up. Bold color and marked ^*∗*^ OR > 1.00 and is statistically significant.

**Table 6:** Effectiveness of follow-up period for global improving allergic rhinitis.

Follow-up period	Study number	OR	*P*	*I* ^2^ (%)	*z*
One month	1	1.26 (0.98, 1.62)	0.072	—	1.8
Three months	4	1.34 (1.17, 1.55)^*∗*^	0.000	0	4.09
Six months	1	1.31 (1.10, 1.57)^*∗*^	0.001	—	3.02
Twelve months	3	1.30 (1.11, 1.53)^*∗*^	0.003	17.8	3.24

Note: ^*∗*^*P* < 0.01.
